# Supplementary material for: TCR-T Cell Recognition of an NY-ESO-1 Epitope Presented by HLA-A2 Supertype: Implications for Cancer Immunotherapy
Source: Vaccines (Basel). 2025 Aug 25;13(9):898. doi: 10.3390/vaccines13090898 (PMC12474311; doi:10.3390/vaccines13090898)
Supplement: Supplementary file 1 [file vaccines-13-00898-s001.zip › vaccines-3766646-supplementary.pdf]

Supplementary Figures and Tables

a

The top 10 frequent HLA-A*02 supertype alleles in Chinese population	Frequency in Chinese population (%)
A*02:01	12.01
A*02:07	8.44
A*02:06	5.21
A*02:03	3.48
A*02:05	0.36
A*02:10	0.35
A*02:53N	0.05
A*02:09	0.037
A*02:11	0.018
A*02:48	0.0097

b

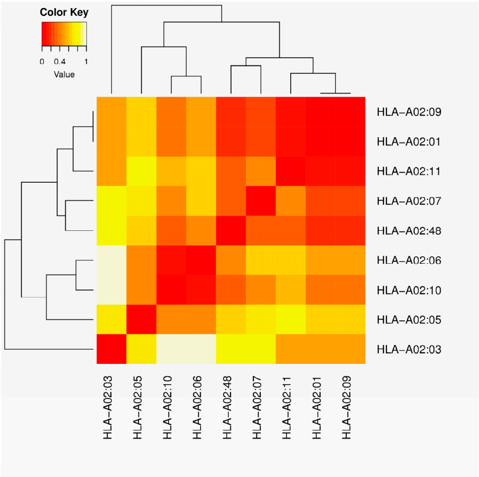

Supplementary Figure 1. Population frequency-based selection of HLA supertype alleles. (a) The top 10 HLA-A2 supertype alleles were chosen based on their frequencies in Chinese population. (b) Functional differences among HLA-A2 supertype alleles based on predicted binding motifs using MHCcluster, HLA-A*02:53N was not included in this analysis because it was a truncated allele.

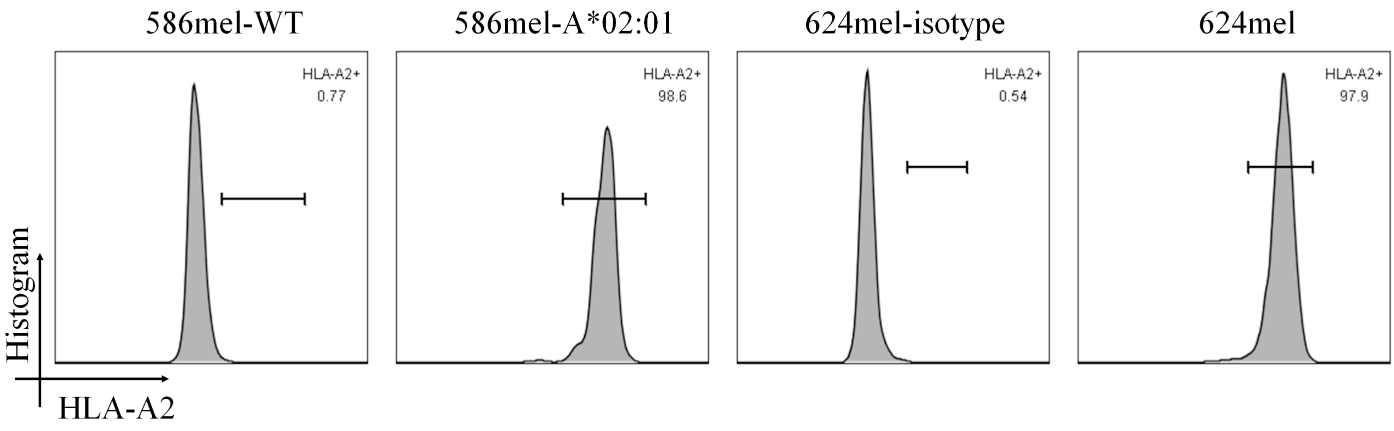

Supplementary Figure 2. Expression of HLA-A*02:01. Expression of HLA-A*02:01 on 586mel-WT, 586mel-A*02:01 and 624mel cells was examined by FACS. For 624mel cells, an isotype control was added for gating.

a

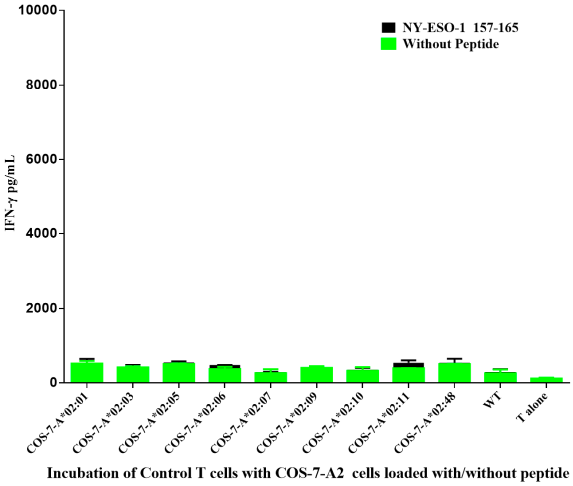

b

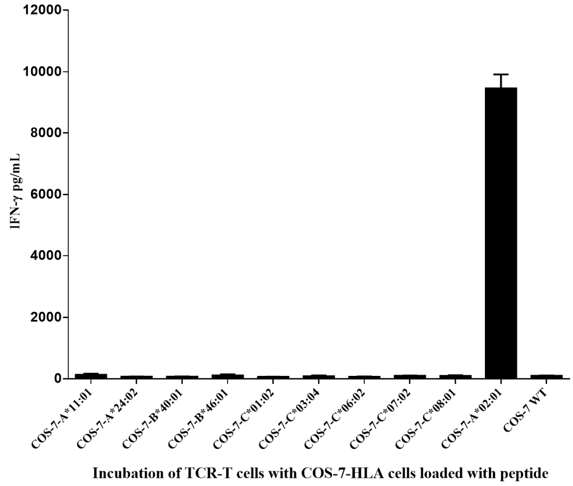

c

Peptide	Sequence	Genbank No.
P1	FLFMWITQR	BAB21793.1
P2	SLLMWLTPL	BAC85512.1
P3	ICLQWITQC	EAW48180.1
P4	LQWITQC	TBC1D32
P5	MWITQR	EAX04473.1
P6	KGLMWISQI	AIZ70768.1
P7	AFLMWITPM	CAD97675.1
P8	QYLMWLTQK	PIK3R1
P9	YLNMWITTC	AAH98167.1
P10	SLLMWMLRL	NEMP2
P11	VLLMWLTAT	EAW68195.1
P12	ALLKWVSDS	BIRC6
P13	GRTMWIHQC	EAW56252.1
P14	SLLLWISGA	CAA77318.1
P15	PLLVWITTL	AAB87902.1
P16	KLDMWITDW	MOM51378.1
P17	SLLVWILTL	KIRREL1
P18	LLLKSVTQA	EAW52854.1

d

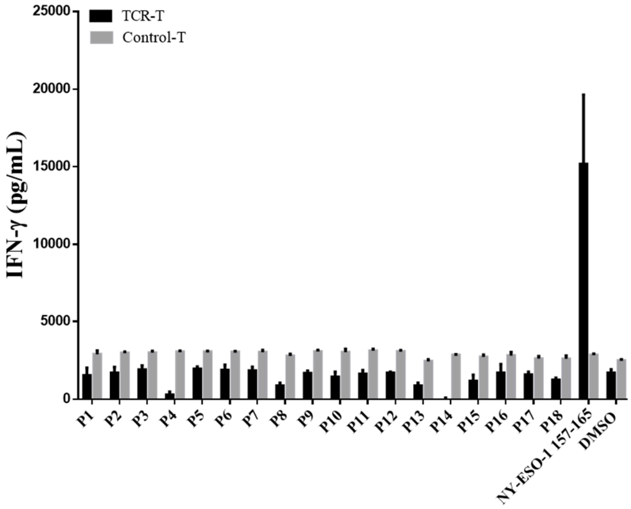

Supplementary Figure 3. Incubation of Control T cells with COS-7-A2 cells and off-target testing of TCR-T cells. (a) IFN-γ secretion results of incubating Control T cells with COS-7-A2 cells loaded with/without peptides. Experiment was performed in triplicate wells and data are representative of four independent experiments (n=4). (b) IFN-γ secretion results of incubating TCR-T cells with COS-7-HLA cells loaded with NY-ESO-1$_{157\text{-}165}$ peptides. Experiment was performed in triplicate wells and data are representative of two independent experiments (n=2). (c) Through sequence alignment with the human protein database (NCBI: non-redundant protein sequence), we screened 18 similar peptides that share at least 3 identical amino acids with the NY-ESO-1$_{157\text{–}165}$ peptide epitope (SLLMWITQC). (d) These peptides were used to pulse T2 cells, which were then co-incubated with TCR-T cells to detect the responses of TCR-T cells to these peptides. Experiment was performed in triplicate wells and data are representative of two independent experiments (n=2). Data are represented as mean ± SD.

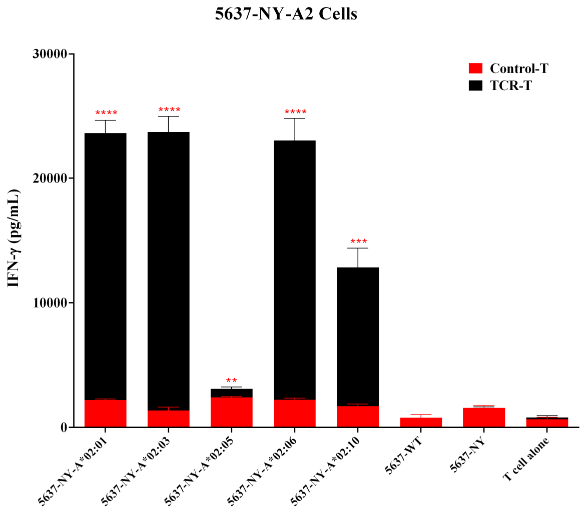

Supplementary Figure 4. HLA-A*02:01 restricted TCR-T cells could recognize target antigen peptide presented by members of the HLA-A2 supertype in 5637 cells. IFN-γ secretion results of incubating TCR-T cells and Control-T cells with 5637-NY-A2 cells. Experiment was performed in triplicate wells and data are representative of four independent experiments (n=4). Data are represented as mean ± SD. *P<0.05, **P<0.01, ***P<0.001 and ****P<0.0001.

a

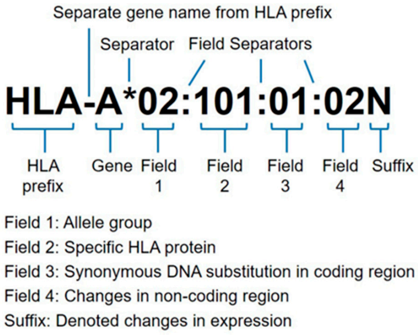

b

```
AA Pos.               -21        -11         -1         10         20         30         40         50         60         70
A*01:01:01:01     MAVM APRTLLLLLS GALALTQTWA GSHSMRYFFT SVSRPGRGEP RFIAVGYVDD TQFVRFDSDA ASQKMEPRAP WIEQEGPEYW DQETRNMKAH
A*02:01:01        ---- -----V---- ---------- ---------- ---------- ---------- ---------- ---R------ ---------- -G---KV---
A*02:03:01        ---- -----V---- ---------- ---------- ---------- ---------- ---------- ---R------ ---------- -G---KV---
A*02:05:01        ---- -----V---- ---------- --------Y- ---------- ---------- ---------- --RR------ ---------- -G---KV---
A*02:06:01        ---- -----V---- ---------- --------Y- ---------- ---------- ---------- ---R------ ---------- -G---KV---
A*02:07:01        ---- -----V---- ---------- ---------- ---------- ---------- ---------- ---R------ ---------- -G---KV---
A*02:09:01        ---- -----V---- ---------- ---------- ---------- ---------- ---------- ---R------ ---------- -G---KV---
A*02:10           ---- -----V---- ---------- --------Y- ---------- ---------- ---------- ---R------ ---------- -G---KV---
A*02:11:01        ---- -----V---- ---------- ---------- ---------- ---------- ---------- ---R------ ---------- -G---KV---
A*02:48           ---- -----V---- ---------- ---------- ---------- ---------- ---------- ---R------ ---------- -E--GKV---

AA Pos.                   80         90        100        110        120        130        140        150        160        170
A*01:01:01:01     SQTDRANLGT LRGYYNQSED GSHTIQIMYG CDVGPDGRFL RGYRQDAYDG KDYIALNEDL RSWTAADMAA QITKRKWEAV HAAEQRRVYL EGRCVDGLRR
A*02:01:01        ---H-VD--- ---------A ----V-R--- ----S-W--- ---H-Y---- ------K--- ---------- -T--H----A -V---L-A-- --T--EW---
A*02:03:01        ---H-VD--- ---------A ----V-R--- ----S-W--- ---H-Y---- ------K--- ---------- -T--H---TA -E---W-A-- --T--EW---
A*02:05:01        ---H-VD--- ---------A ----L-R--- ----S-W--- ---H-Y---- ------K--- ---------- -T--H----A -V---W-A-- --T--EW---
A*02:06:01        ---H-VD--- ---------A ----V-R--- ----S-W--- ---H-Y---- ------K--- ---------- -T--H----A -V---L-A-- --T--EW---
A*02:07:01        ---H-VD--- ---------A ----V-R-C- ----S-W--- ---H-Y---- ------K--- ---------- -T--H----A -V---L-A-- --T--EW---
A*02:09:01        ---H-VD--- ---------A ----V-R--- ----S-W--- ---H-Y---- ------K--- ---------- -T--H----A -V---L-A-- --T--EW---
A*02:10           ---H-VD--- ---------A ----V-R-F- ----S----- ---H-Y---- ------K--- ---------- -T--H----A -V---L-A-- --T--EW---
A*02:11:01        --I--VD--- ---------A ----V-R--- ----S-W--- ---H-Y---- ------K--- ---------- -T--H----A -V---L-A-- --T--EW---
A*02:48           -----VD--- ---------A ----V-R--- ----S-W--- ---H-Y---- ------K--- ---------- -T--H----A -V---L-A-- --T--EW---

AA Pos.                  180        190        200        210        220        230        240        250        260        270
A*01:01:01:01     YLENGKETLQ RTDPPKTHMT HHPISDHEAT LRCWALGFYP AEITLTWQRD GEDQTQDTEL VETRPAGDGT FQKWAAVVVP SGEEQRYTCH VQHEGLPKPL
A*02:01:01        ---------- ---A------ --AV------ ------S--- ---------- ---------- ---------- ---------- --Q------- ----------
A*02:03:01        ---------- ---A------ --AV------ ------S--- ---------- ---------- ---------- ---------- --Q------- ----------
A*02:05:01        ---------- ---A------ --AV------ ------S--- ---------- ---------- ---------- ---------- --Q------- ----------
A*02:06:01        ---------- ---A------ --AV------ ------S--- ---------- ---------- ---------- ---------- --Q------- ----------
A*02:07:01        ---------- ---A------ --AV------ ------S--- ---------- ---------- ---------- ---------- --Q------- ----------
A*02:09:01        ---------- ---A------ --AV------ ------S--- ---------- ---------- -----E---- ---------- --Q------- ----------
A*02:10           ---------- ---A------ --AV------ ------S--- ---------- ---------- ---------- ---------- --Q------- ----------
A*02:11:01        ---------- ---A------ --AV------ ------S--- ---------- ---------- ---------- ---------- --Q------- ----------
A*02:48           ---------- ---A------ --AV------ ------S--- ---------- ---------- ---------- ---------- --Q------- ----------

AA Pos.                  280        290        300        310        320        330        340
A*01:01:01:01     TLRWELSSQP TIPIVGIIAG LVLLGAVITG AVVAAVMWRR KSSDRKGGSY TQAASSDSAQ GSDVSLTACK V
A*02:01:01        -----P---- ---------- ---F------ ---------- ---------- S--------- ---------- -
A*02:03:01        -----P---- ---------- ---F------ ---------- ---------- S--------- ---------- -
A*02:05:01        -----P---- ---------- ---F------ ---------- ---------- S--------- ---------- -
A*02:06:01        -----P---- ---------- ---F------ ---------- ---------- S--------- ---------- -
A*02:07:01        -----P---- ---------- ---F------ ---------- ---------- S--------- ---------- -
A*02:09:01        -----P---- ---------- ---F------ ---------- ---------- S--------- ---------- -
A*02:10           -----P---- ---------- ---F------ ---------- ---------- S--------- ---------- -
A*02:11:01        -----P---- ---------- ---F------ ---------- ---------- S--------- ---------- -
A*02:48           -----P---- ---------- ---F------ ---------- ---------- S--------- ---------- -
```

Supplementary Figure 5. Alignment of HLA-A2 alleles. (a) The different fields used in standard HLA nomenclature (1). (b) Because Field 3 and Field 4 didn't affect protein sequences of HLA alleles, we chose one subtype of each allele to be analyzed by HLA Sequence Alignment: A FULLPROTEIN - Release 3.57.0 (2024-07-08) (https://www.ebi.ac.uk/cgi-bin/ipd/pl/hla/align.cgi). Mismatches were highlighted. The start codon of the mature protein is labeled codon 1. The codon 5' to this is numbered -1. Note: that there is no amino acid 0.

Reference

1. Kishore A, Petrek M. Next-Generation Sequencing Based HLA Typing: Deciphering Immunogenetic Aspects of Sarcoidosis. Front Genet. 2018;9:503.

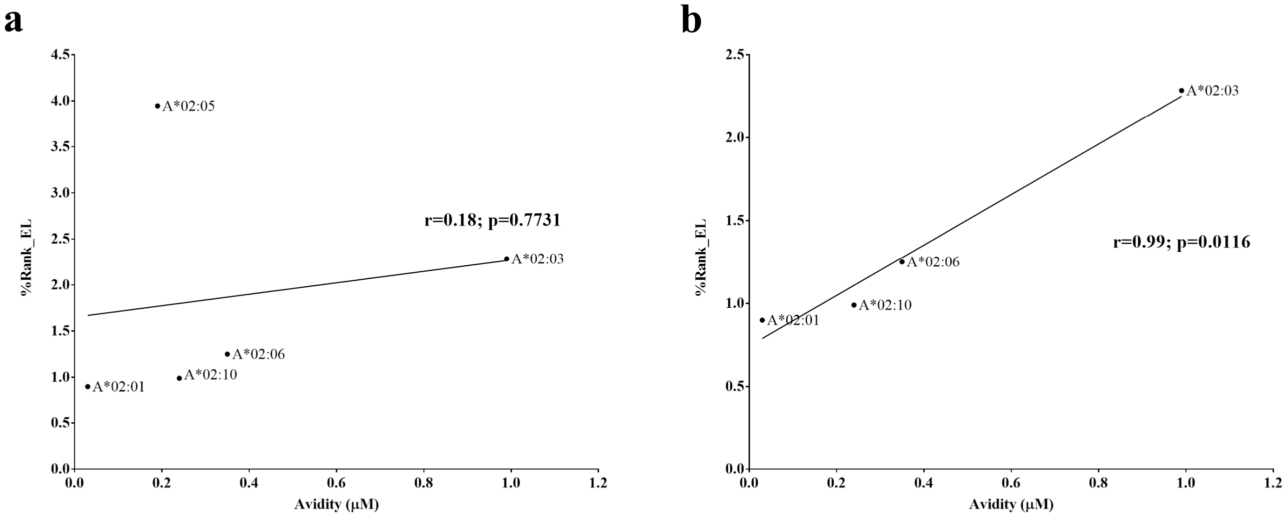

Supplementary Figure 6. Correlation between the avidity and predicted binding affinity. Correlation between the avidity and predicted binding affinity of epitopes with HLA-A2 alleles by NetMHCpan 4.1. HLA-A*02:05 was included in (a) and excluded in (b). The statistical analysis was performed using Pearson r test of Correlation with GraphPad Prism 6.01 software and the P<0.05 was considered to indicate a statistically significant difference.

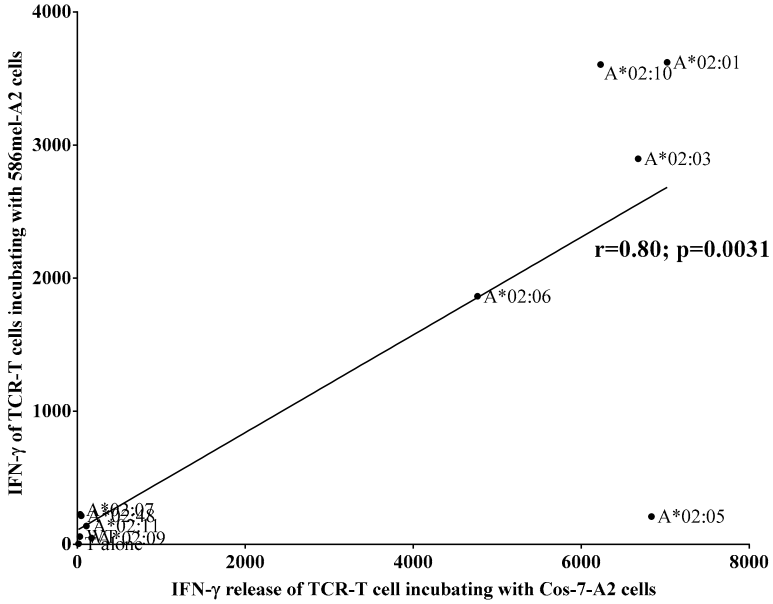

Supplementary Figure 7. Functional correlation between the exo- and endogenously presented epitopes. Correlation between the activities of COS-7-A2 and 586mel-A2 cells according to both ELISA. The statistical analysis was performed using Pearson r test of Correlation with GraphPad Prism 6.01 software and the P<0.05 was considered to indicate a statistically significant difference.

Supplementary Table 1. The percentage and MFI of HLA-A2-positive cells

HLA alleles	HLA-A2+ Cells			
	COS-7-A2		586mel-A2	
	Percentage	MFI	Percentage	MFI
NC	0	n/a	0	n/a
HLA-A*02:01	90.5	185744	98.6	300224
HLA-A*02:03	94	270062	99.5	314725
HLA-A*02:05	64.7	147710	98.1	214358
HLA-A*02:06	67.9	185257	97.8	226738
HLA-A*02:07	63.3	89184	92.7	111327
HLA-A*02:09	69.4	93636	96.7	95836
HLA-A*02:10	0	n/a	0.8	n/a
HLA-A*02:11	52.1	191785	97.7	241450
HLA-A*02:48	67.4	141476	95.2	161078
HLA-A*02:53	0.38	n/a	0.96	n/a
WT	0	n/a	0.77	n/a

Supplementary Table 2. Expression of transduced HLA-A2 and NY-ESO-1 genes

Cell		Percentage of A2+ cells	Percentage of NY-ESO-1+ cells	Percentage of A2+ NY-ESO-1+ cells
COS-7-NY-A2 cell	WT	0	0	0
	NY	0	94.8	0
	NY-HLA-A*02:01	98.5	97.3	94.8
	NY-HLA-A*02:03	98.3	96.2	92.8
	NY-HLA-A*02:05	87.6	95.8	86.3
	NY-HLA-A*02:06	91.8	94.8	87.4
	NY-HLA-A*02:10	n/a	94.7	n/a
5637-NY-A2 cell	WT	0	0	0
	NY-HLA-A*02:01	98.0	96.0	96.2
	NY-HLA-A*02:03	63.4	81.0	49.2
	NY-HLA-A*02:05	64.4	52.8	35.1
	NY-HLA-A*02:06	64.0	78.1	47.5
	NY-HLA-A*02:10	n/a	60.5	n/a

Supplementary Table 3. Summary of mismatches of HLA-A2 alleles' sequence

HLA-A2 alleles	AA Pos.[a]								Avidity (μM)
	Alpha-1				Alpha-2			Alpha-3	
	9	43	65	73	99	149	156	236	
**HLA-A*02:01**	F	Q	R	T	Y	A	L	A	0.03
**HLA-A*02:03**	-	-	-	-	-	T	W	-	0.99
**HLA-A*02:05**	Y	R	-	-	-	-	W	-	0.19
**HLA-A*02:06**	Y	-	-	-	-	-	-	-	0.35
HLA-A*02:07	-	-	-	-	C	-	-	-	/
HLA-A*02:09	-	-	-	-	-	-	-	E	/
**HLA-A*02:10**	Y	-	-	-	F	-	-	-	0.24
HLA-A*02:11	-	-	-	I	-	-	-	-	/
HLA-A*02:48	-	-	G	-	-	-	-	-	/

a. "-" means similar amino acid in the position

Supplementary Table 4. Regions of HLA-A

AA Pos. [a]		Region	Function
Start	End		
-22	-14	VL9 epitope	Signal sequence
1	90	Alpha-1	Peptide-binding cleft
91	182	Alpha-2	Peptide-binding cleft;<br>Mediates the interaction with TAP1-TAP2 complex
183	274	Alpha-3	The alpha-3 Ig-like domain (185-271) mediates the interaction with CD8 co-receptor
275	284	Connecting peptide	/
315	341	Disordered	/

a. AA Pos. is transformed to be consistent with the format of alignment

Supplementary Table 5. MHC-I binding and stability prediction results

HLA-A2 alleles	Peptide	NetMHCpan 4.1	NetMHCstabpan 1.0	
		%Rank_EL[a]	Half-lives(h)	%Rank_Stab BindLevel [b]
**HLA-A*02:01**	SLLMWITQC	0.899	2.41	1.60
**HLA-A*02:03**		2.285	0.94	3.00
**HLA-A*02:05**		3.946	0.82	4.00
**HLA-A*02:06**		1.250	1.31	2.50
HLA-A*02:07		1.824	1.03	1.80
HLA-A*02:09		0.899	2.41	1.60
**HLA-A*02:10**		0.990	0.59	3.00
HLA-A*02:11		0.632	2.43	1.50
HLA-A*02:48		1.320	0.91	1.70

a. High binders (%Rank_EL<0.5), weak binders (%Rank_EL<2)

b. High binders (%Rank_Stab BindLevel<0.5), weak binders (%Rank_Stab BindLevel<2)

Supplementary Table 6. Predominant HLA-A2 Alleles among different populations

Country	USA				German		India		Japan	
Population	USA NMDP African American pop 2		USA NMDP European Caucasian		Germany DKMS - German donors		India South UCBB		Japan pop 16	
Sample Size	416,581		1,242,890		3,456,066		11,446		18,604	
Predominant HLA-A2 Alleles	Alleles	Frequency (%)	Alleles	Frequency (%)	Alleles	Frequency (%)	Alleles	Frequency (%)	Alleles	Frequency (%)
	**A*02:01**	**12.35**	**A*02:01**	**27.55**	**A*02:01**	**28.39**	A*02:11	9.59	**A*02:01**	**11.62**
	A*02:02	4.14	A*02:05	0.97	A*02:05	0.7	**A*02:01**	**4.67**	**A*02:06**	**9.08**
	A*02:05	1.49	**A*02:06**	**0.18**	**A*02:06**	**0.19**	**A*02:06**	**1.65**	A*02:07	3.46
	**A*02:06**	**0.07**	A*02:02	0.09	A*02:17	0.1	**A*02:03**	**1.24**	**A*02:10**	**0.39**
	A*02:60	0.05	A*02:17	0.04	A*02:02	0.06	A*02:09	0.61	A*02:18	0.08
	A*02:11	0.02	A*02:20	0.01	A*02:30	0.06	A*02:131	0.55	**A*02:03**	**0.06**
	A*02:17	0.02	A*02:24	0.01	A*02:08	0.02	A*02:05	0.35	A*02:05	0.01
	**A*02:03**	**0.02**	A*02:22	0.01	A*02:07	0.02	A*02:20	0.04	A*02:13	0.01
	A*02:22	0.01	A*02:11	0.01	A*02:35	0.02	A*02:273	0.01	A*02:28	0.01
	A*02:14	0.01	/	/	A*02:22	0.02	A*02:744	0.01	A*02:53N	0.01
